# Effects of concentration and chain length of the sequence copolymer on interfacial properties of homopolymers/sequence copolymers ternary blends: A DPD simulation study

**DOI:** 10.1371/journal.pone.0270094

**Published:** 2022-07-26

**Authors:** Dongmei Liu, Huifeng Bo, Yongchao Jin, Deyang Li, Zhanxin Zhang, Kai Gong, Ye Lin, Sijia Li

**Affiliations:** 1 School of Science, North China University of Science and Technology, Tangshan, P. R. China; 2 School of Intelligence Policing, People’s Police University of China, Langfang, P. R. China; Rice University, UNITED STATES

## Abstract

The effect of the concentration and chain length of the copolymer AB with sequence length *τ* = 8 on the interfacial properties of the ternary mixtures A_10_/AB/B_10_ are investigated by the dissipative particle dynamics (DPD) simulations. It is found that: i) As the copolymer concentration varies from 0.05 to 0.15, increasing the copolymer enrichment at the center of the interface enlarges the interface width *ω* and reduces the interfacial tension. However, as the concentration of the sequence copolymers further increases to 0.2, because the interface has formed micelles and the micellization could lower the efficiency of copolymers as a compatibilizer, the interfacial tension exhibits a slightly increase; ii) elevating the copolymer chain length, the copolymer volumes vary from a cylinder shape to a pancake shape. The blends of the copolymer with chain length *N*_cp_ = 24 exhibit a wider interfacial width *w* and a lower interfacial tension *γ*, which indicates that the sequenced copolymer *N*_cp_ = 24 exhibits a better performance as the compatibilizers. This study illustrates the correlations between the reduction in interfacial tension produced by the sequence copolymers and their molecular parameters, which guide a rational design of an efficient compatibilizer.

## Introduction

The interfacial behavior of copolymers AB between phase-separated homopolymers A and B has long been the focus of research [1]. It was shown in previous studies that block copolymers tend to aggregate at the interface [1–8]. The aggregated block copolymers can compatibilized the immiscible homopolymers and improve the mechanical strength of the composite materials. Though considerable attention has been focused on the simplest diblock copolymer compatibilizers during the last three decades, some research found that the compatibility of a copolymer strongly correlates to the architecture and sequence distribution of copolymers and, thereby, impacts the ability of a copolymer to strengthen the interface [9].

Both theoretical [10, 11] and experimental [3, 9] studies have proven that as the sequence distribution of a copolymer varies from alternating copolymer to diblock, the ability of the copolymer to modify a phase separation interface will also change. When the diblock copolymers are used to compatibilized the immiscible homopolymers, they will form a cylindrical or dumbbell shape. As the sequence length of the copolymer decreases under the fixed copolymer chain length, the time of the copolymer crossing the interface will increase. As the sequence length of the copolymer further decreases and becomes alternating, the copolymer will cover a substantial area of the interface and attain a pancake-type structure. The number of times the alternating copolymer crosses the interface and the degree of penetration into the homopolymer phase are far less than that of the diblock copolymers [9].

Computer simulations provide another way to probe the structural and interfacial properties of the ternary mixtures comprised of sequence copolymers. Ko and Jo [12] found that the copolymer of different architectures exhibited different conformation by a Monte Carlo simulation. That is, the diblock copolymer chains spanned across the interface once and each block extended to the corresponding homopolymer phases, the random copolymers weaved back and forth across the interface [12], whereas the alternating copolymers only lay on the interface and hardly extended to the homopolymer phases. Dadmun [12–15] employed Monte Carlo simulations to explore the structure and miscibility of a ternary blend with different copolymer architectures. They found that though both block and alternating copolymers can modify the interface, the purely random copolymer had the weakest effect on interfacial strengthening. Genzer and coworkers [16] compared the effectiveness of block, alternating, random copolymers and protein-like copolymers (PLCs) at low copolymer concentration (0.66%) using discontinuous molecular dynamics (DMD) computer simulations. It was found that as efficient interfacial compatibilizers, PLCs with high-molecular-weight are likely to outperform the random or alternating copolymers. Subsequently, they explored the dynamics of the phase separation in the blends of A/PLC/B by kinetic Monte Carlo simulation, which showed that the PLC not only could effectively slow down the process of phase separation but also minimize the unfavorable components contacts, thereby reducing the interfacial tension [17]. In a recent paper [18] an investigation was presented on the interfacial and structural properties for ternary mixtures of copolymers with different sequence distributions by dissipative particle dynamics (DPD) simulations. The simulations showed that the interfacial tension decreased by increasing the sequence length of the copolymer from *τ* = 2 (the alternating structure) to 8, whereas it increased by further increasing the sequence length of the copolymer from *τ* = 8 to 32 (the diblock), thus the copolymer with sequence length *τ* = 8 was more effective in reducing the interfacial tension. We defined *τ* = 2 to correspond to an alternating structure and *τ* = 32 to a diblock referred to the studies of Genzer et al. [16] and Chang et al. [19]. Therefore, it would be necessary to further systematically study the effects of other molecular parameters (e.g. the concentration and the chain length of the copolymer) on the interfacial and structural properties of the interfaces for the ternary mixture composed of copolymer with sequence length *τ* = 8.

In the present paper, the earlier work is extended by investigating the effect of the concentration and length of copolymer with a sequence length *τ* = 8. DPD simulations are employed to study the interfacial and structural properties of ternary blends composed of copolymers with sequence length *τ* = 8. Below, we first describe the DPD methods and the simulation details of this work. Then, in the following section, we show the simulation results, and systemically analyze and discuss the results. We highlight the important role of the sequence copolymer concentration in reducing interfacial tension. Further, we elucidate the fundamental mechanism that the copolymers AB(*τ* = 8) with the chain length *N*_cp_ = 24 for strengthing the interface is more prominent. Finally, a brief summarize and some concluding remarks are offered.

## Method

Dissipative particle dynamics (DPD) is a coarse-grained method presenting two important features [20]. First, in DPD simulation a bead usually represents a whole molecule or a molecule fragment. Second, the interaction between different beads occurs through soft potential, which results in the beads can overlap considerably. These features allow studying the structural and dynamic properties of polymer systems in a much larger length and time scale, which hardly be accessed by the all-atom molecule dynamics simulations [21]. These features also allow DPD to efficiently study the highly dispersed polymer mixtures. The model of this paper was constructed based on the previous studies of dispersed polymeric mixtures [18, 20, 22–37], which were briefly introduced as follows.

### Model

The motion of all DPD beads abides by Newton’s second law [38],

$$\begin{array}{c} \frac{dr_{i}}{dt}=v_{i};m_{i}\frac{dv_{i}}{dt}=F_{i} \end{array}$$ (1)

where *r*_*i*_, *v*_*i*_, *m*_*i*_ represent the position vector, velocity vector, and mass of bead *i*, respectively. For the convenience of calculation, the mass *m*_*i*_ is taken as 1. The total external force **F**_*i*_ exerting on bead *i* can be divided into four component forces, which are the conservative force $\left(F_{ij}^{C}\right)$, the dissipative force $\left(F_{ij}^{D}\right)$, the random force $\left(F_{ij}^{R}\right)$ and the spring force $\left(F_{i}^{S}\right)$. The total force acting on *i*th bead by other beads and the component forces are given by:

$$\begin{array}{c} F_{i}=\sum_{j\neq i}(F_{ij}^{C}+F_{ij}^{D}+F_{ij}^{R})+F_{i}^{S} \end{array}$$ (2)

$$\begin{array}{c} F_{ij}^{C}=\{\begin{array}{cc} \alpha_{\text{AB}}(1-r_{ij})e_{ij} & \left(r_{ij}<1\right)\\ 0 & \left(r_{ij}\geq 1\right) \end{array} \end{array}$$ (3)

$$\begin{array}{c} F_{ij}^{D}=\{\begin{array}{cc} -\gamma \omega^{D}(r_{ij})(v_{ij}\cdot e_{ij})e_{ij} & \left(r_{ij}<1\right)\\ 0 & \left(r_{ij}\geq 1\right) \end{array} \end{array}$$ (4)

$$\begin{array}{c} F_{ij}^{R}=\{\begin{array}{cc} \sigma \omega^{R}(r_{ij})\xi_{ij}\Delta t^{1/2}e_{ij} & \left(r_{ij}<1\right)\\ 0 & \left(r_{ij}\geq 1\right) \end{array} \end{array}$$ (5)

$$\begin{array}{c} F_{i}^{S}=\sum_{j\neq i}Cr_{ij} \end{array}$$ (6)

where *α*_AB_ is the repulsive parameter. *r*_*ij*_ = *r*_*i*_ − *r*_*j*_, *r*_*ij*_ = |*r*_*ij*_|, *e*_*ij*_ = *r*_*ij*_/*r*_*ij*_ is the unit vector of *r*_*ij*_. *r*_*ij*_ represents the distance between beads *i* and *j*; *γ* is the friction coefficient of dissipative force; *σ* is the nose amplitude, which determines the intensity of the random; *ξ*_*ij*_ is a Gaussian random number. To ensure that the simulation obeys a canonical ensemble, the weight functions *ω*^D^(*r*_*ij*_) and *ω*^R^(*r*_*ij*_) for the dissipative and random processes, as well as the friction coefficient *γ* and the nose amplitude *σ* followed the fluctuation-dissipation theorem [38].

$$\begin{array}{c} \sigma_{ij}^{2}=2\gamma k_{B}T \end{array}$$ (7)

$$\begin{array}{c} \omega^{D}(r_{ij})={\left[\omega^{R}(r_{ij})\right]}^{2}=\{\begin{array}{cc} {\left(1-r_{ij}\right)}^{2} & \left(r_{ij}<1\right)\\ 0 & \left(r_{ij}\geq 1\right) \end{array} \end{array}$$ (8)

where *k*_B_*T* = 1, *k*_B_ and *T* represents the Boltzmann constant and temperature.

The repulsive parameter *α*_AB_ can be calculated from the Flory-Huggins parameter *χ*_*AB*_ according to expression [38].

$$\begin{array}{c} \alpha_{\text{AB}}\approx \alpha_{\text{AA}}+3.27\chi_{\text{AB}} \end{array}$$ (9)

The repulsive parameters between the same and the different types of beads are *α*_AA_ = *α*_BB_ = 25 and *α*_AB_ = 40, respectively [28].

### Simulation details

Materials Studio was used as the simulator of the present work. All simulations were carried out in a 30 × 30 × 30 cubic box in DPD reduced units with the number density *ρ* = 3, thus the system contains approximately 81000 beads. Periodic boundary conditions are applied in all three directions. The interaction radius and the friction coefficient are set as *r*_*c*_ = 1 and *γ* = 4.5 in DPD reduced units respectively. To ensure that the simulations reach the equilibration state, 2.0 × 10^5^ time steps are first performed with the time step Δ*t* = 0.05 in DPD reduced units [39]. After that, 5.0 × 10^4^ time steps are performed. To achieve good statistics, we averaged the data of 10^3^ − 10^4^ independent samples from 5 parallel simulation runs.

**Fig 1:**
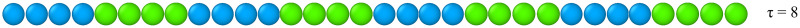
Schematic for copolymer AB of the sequence length *τ* = 8 and chain length *N*_cp_ = 32, where the blue and green spheres denote beads A and B of copolymer respectively.

In this paper, we focus on the mixtures composed of homopolymer A_10_, B_10_, and copolymer AB with sequence length *τ* = 8. The sequence length *τ* = 8 is defined by its periodicity, e.g. if a copolymer has an architecture that 4 A beads followed by 4 B beads, the sequence length of the copolymer is *τ* = 8. The content of homopolymers A_10_ and B_10_ are equal. In order to investigate the effect of the sequence copolymer concentration on the interfacial properties, we vary copolymer concentration from *c*_cp_ = 0.01 to 0.2 (*c*_cp_ = the number of copolymer beads / the total number of beads) while the chain length and the sequence length of the copolymer were fixed as of *N*_cp_ = 32 and *τ* = 8 respectively. The concentration here represents the volume fraction of the sequence copolymer AB in the simulation system. Fig 1 shows the schematic of AB copolymers of *τ* = 8, *N*_cp_ = 32. To explore the effect of sequence copolymer AB(*τ* = 8) chain length on the interfacial properties, the chain length of the copolymers are set as *N*_cp_ = 8, 16, 24, 32, 48, 64 with the copolymer concentration *c*_cp_ = 0.15.

Interfacial tension is an important parameter to measure the properties of the polymeric blend system. In the conditions of the interface is perpendicular to the x-axis, the interfacial tension can be calculated following the Irving-Kirkwood equation [40]

$$\begin{array}{c} \gamma_{DPD}=\frac{1}{2}L[\langle P_{xx}\rangle -\frac{1}{2}\left(\langle P_{yy}\rangle +\langle P_{zz}\rangle \right)] \end{array}$$ (10)

where *P*_*xx*_, *P*_*yy*_, *P*_*zz*_ represent the pressure tensor of the *x*, *y*, and *z*-direction respectively, <> denotes the ensemble average.

To explore the microscopic configurations of the copolymers, we calculate the mean-square radius of gyration $\langle R_{g}{}^{2}\rangle$ and its three components ${\langle R_{g}{}^{2}\rangle }_{x}$, ${\langle R_{g}{}^{2}\rangle }_{y}$ and ${\langle R_{g}{}^{2}\rangle }_{z}$, and the orientation parameter *q* of the sequence copolymers. ${\langle R_{g}{}^{2}\rangle }_{x}$ is the component normal to the interface, ${\langle R_{g}{}^{2}\rangle }_{y}$ and ${\langle R_{g}{}^{2}\rangle }_{z}$ are the components parallel to the interface. The orientation parameter *q* is obtained by [28]

$$\begin{array}{c} q=\frac{\left({\left\langle R_{g}^{2}\right\rangle }_{x}-1/2\left({\left\langle R_{g}^{2}\right\rangle }_{y}+{\left\langle R_{g}^{2}\right\rangle }_{z}\right)\right)}{\left\langle R_{g}^{2}\right\rangle } \end{array}$$ (11)

Additionally, we calculate the interfacial width *w* by fitting the function tanh ((*x* + *d*)/*w*) to the (*ρ*^*A*^(*x*) − *ρ*^*B*^(*x*))/*ρ*(*x*) across the interface, where *d* is the shift of the interface center along the *x*-axis [29].

## Results and discussion

### Effects of the sequence copolymer AB(*τ* = 8) concentration

In this section, the influence of the concentration of the sequence copolymer AB on the interfacial properties is discussed. To accelerate the formation of interfaces, the homopolymers A_10_, B_10_, and copolymers AB(*τ* = 8) were initially placed in distinct positions of the *x*-direction in the box. The copolymer concentration is set as *c*_cp_ = 0.05, 0.1, 0.15 and 0.2 while the chain length and the sequence length of the copolymers were *N*_cp_ = 32 and *τ* = 8 respectively.

**Fig 2:**
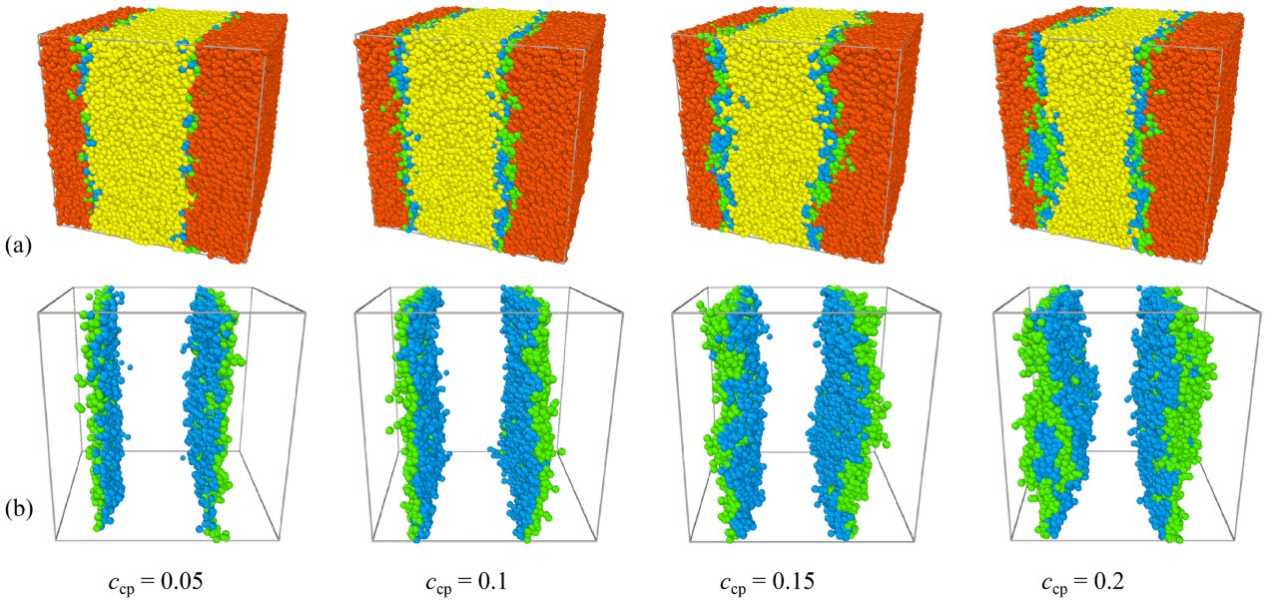
Morphology snapshots of systems at different sequence copolymer concentrations. (a)A_10_/AB(*τ* = 8)/B_10_ ternary blends and (b) AB(*τ* = 8) copolymers at *N*_cp_ = 32. The red and yellow spheres denote bead A and bead B of homopolymers A_10_ and B_10_, and the green and blue spheres represent beads A and B of the AB copolymers.

**Fig 3:**
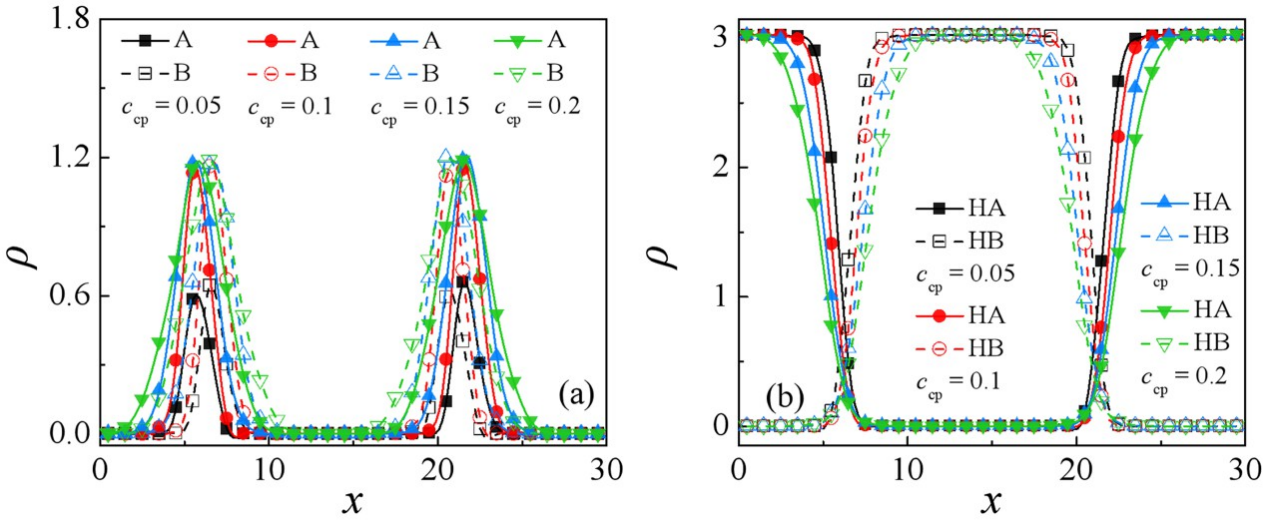
Density profiles of beads A and B of the (a) AB(*τ* = 8) copolymers and (b) A_10_ and B_10_ homopolymers in the *x*-direction as a function of the copolymer AB(*τ* = 8) concentration at *N*_cp_ = 32.

Figs 2 and 3 show the morphology snapshots and density profiles for the blends of A_10_/AB(*τ* = 8)/ B_10_ of different copolymer concentrations, respectively. It is found that as the copolymer concentration increases from *c*_cp_ = 0.05 to 0.2, the enrichment of the copolymers at the interfaces exhibits significant increases [Figs 2 and 3(a)], which results in a decayed correlation between the *A*_10_ and *B*_10_ homopolymers, as shown in Fig 3(b). Specifically, as the concentration of the sequence copolymer AB(*τ* = 8) increases from *c*_cp_ = 0.05 to 0.1, the density of the beads A and B of the copolymers at the interfaces increases [the black squares and red dots in Fig 3(a)], whereas as *c*_cp_ of the sequence copolymer AB(*τ* = 8) further increases to 0.15 and 0.2, the density of the beads A and B of the copolymers at the center of the interfaces almost unchanged, the distribution widths of beads A and B of copolymers broaden. It is noticeable that as *c*_cp_ = 0.15 and 0.2, the interfaces have reached saturated: i) at *c*_cp_ = 0.15, the interfaces curved, ii) at *c*_cp_ = 0.2, the copolymers formed micelles at the interface. To investigate the effect of initial positions, we simulated the evolution of the blends’ morphology with all the chain positions that were initially randomized, which is shown in S1 Fig. S1 Fig shows that as the system did not reach equilibrium *t* = 50000, some copolymers dissolved in the bulk, whereas as the system reached equilibrium *t* = 200000, the superfluous copolymers also form micelles at the interface instead of dissolving in the bulk. We infer that this result is related to the energy change in the system.

**Fig 4:**
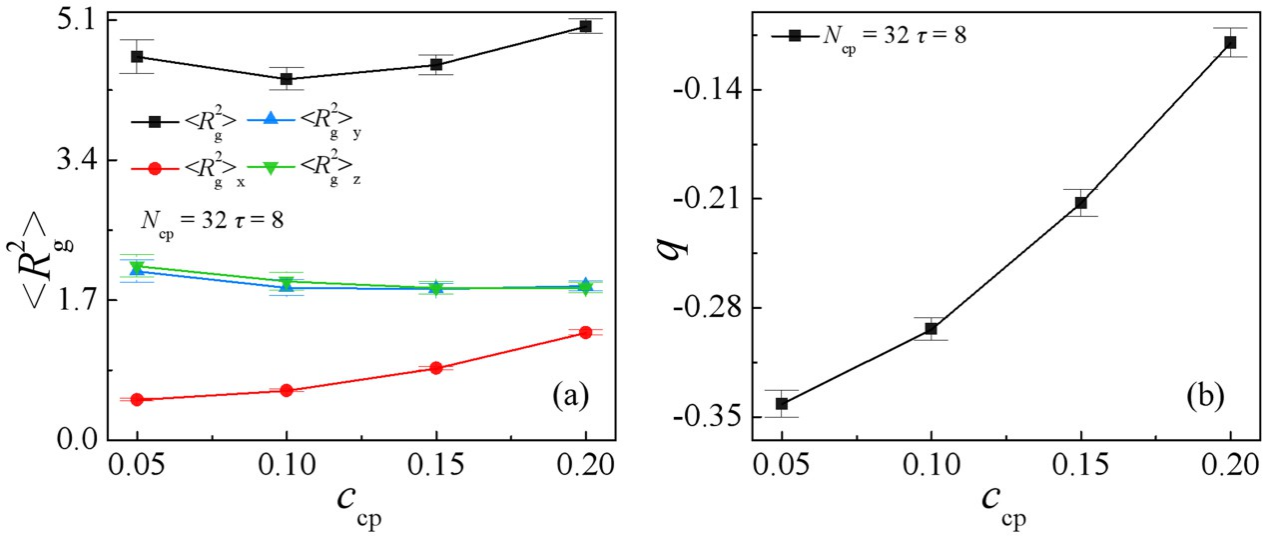
Mean-square radii of gyration $\langle R_{g}{}^{2}\rangle$ and the three components ${\langle R_{g}{}^{2}\rangle }_{x}$, ${\langle R_{g}{}^{2}\rangle }_{y}$ and ${\langle R_{g}{}^{2}\rangle }_{z}$ (a), and orientation parameter *q* (b) of copolymers as a function of the copolymer AB(*τ* = 8) concentration at *N*_cp_ = 32.

We calculate the mean-square radius of gyration $\langle R_{g}{}^{2}\rangle$ and its three components ${\langle R_{g}{}^{2}\rangle }_{x}$,${\langle R_{g}{}^{2}\rangle }_{y}$ and ${\langle R_{g}{}^{2}\rangle }_{z}$, the orientation parameter *q* of the sequence copolymers AB(*τ* = 8) at different copolymer concentrations, as shown in Fig 4(a) and 4(b). It is found that as the sequence copolymers AB(*τ* = 8) concentration increases from *c*_cp_ = 0.05 to 0.2, ${\langle R_{g}{}^{2}\rangle }_{x}$ exhibits an obvious increase, whereas ${\langle R_{g}{}^{2}\rangle }_{y}$ and ${\langle R_{g}{}^{2}\rangle }_{z}$ decrease slightly, which results in an increase of the orientation parameter *q* [Fig 4(b)]. Because of that ${\langle R_{g}{}^{2}\rangle }_{y}$ and ${\langle R_{g}{}^{2}\rangle }_{z}$ are greater than ${\langle R_{g}{}^{2}\rangle }_{x}$, the orientation parameter *q* < 0, which indicates that the copolymer AB with the sequence length *τ* = 8 is mainly oriented along with the directions of parallel to the interfaces.

**Fig 5:**
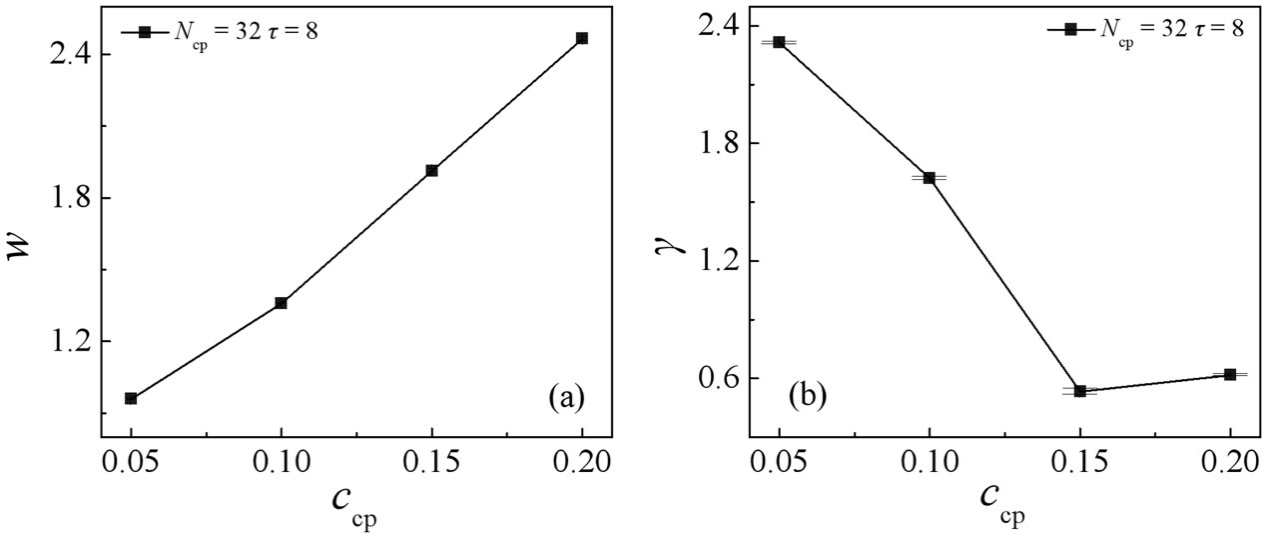
Interfacial width *ω* (a), and the interfacial tension *γ* (b) of the blends as a function of the copolymer AB(*τ* = 8) concentration at *N*_cp_ = 32.

The variations of the interfacial width *ω* and the interfacial tension *γ* in the ternary blends with increasing the sequence copolymers AB(*τ* = 8) concentration *c*_cp_ are shown in Fig 5(a) and 5(b). As expected, the interfacial width *ω* increases monotonically with increasing the sequence copolymers AB(*τ* = 8) concentration *c*_cp_, which is caused by the increase of the enrichment of the copolymers at the interfaces. However, the interfacial tension *γ* decreases fast first and then increases slightly with increasing the sequence copolymers AB(*τ* = 8) concentration *c*_cp_. Specifically, with increasing the sequence copolymers AB(*τ* = 8) concentration *c*_cp_ from 0.05 to 0.15, a remarkable drop in the interfacial tension is observed, this is because as *c*_cp_ increases from 0.05 to 0.15, the density of beads A and B of the sequence copolymers AB(*τ* = 8) at the interface increases [as illustrated in Figs 2 and 3(a)], which results in the decayed correlations between the A_10_ and B_10_ homopolymer, thus the interfacial tension *γ* decreases. Further, as *c*_cp_ increases from 0.15 to 0.2, the interfacial tension *γ* increases slightly. A possible explanation for the elevatory interfacial tension at the concentration *c*_cp_ = 0.2 may be the result of the formation of the micelles [Fig 2 *c*_cp_ = 0.2]. The micellization can lower the efficiency of the copolymer as compatibilizers [41], therefore, the interfacial tension *γ* increases slightly.

### Effects of the sequence copolymer AB (*τ* = 8) chain length

To investigate how the chain length of the copolymer AB (*τ* = 8) influences the compatibilization of the phase-separated, we vary the chain length of the copolymers AB (*τ* = 8) from *N*_cp_ = 8 to *N*_cp_ = 64, at *c*_cp_ = 0.15.

**Fig 6:**
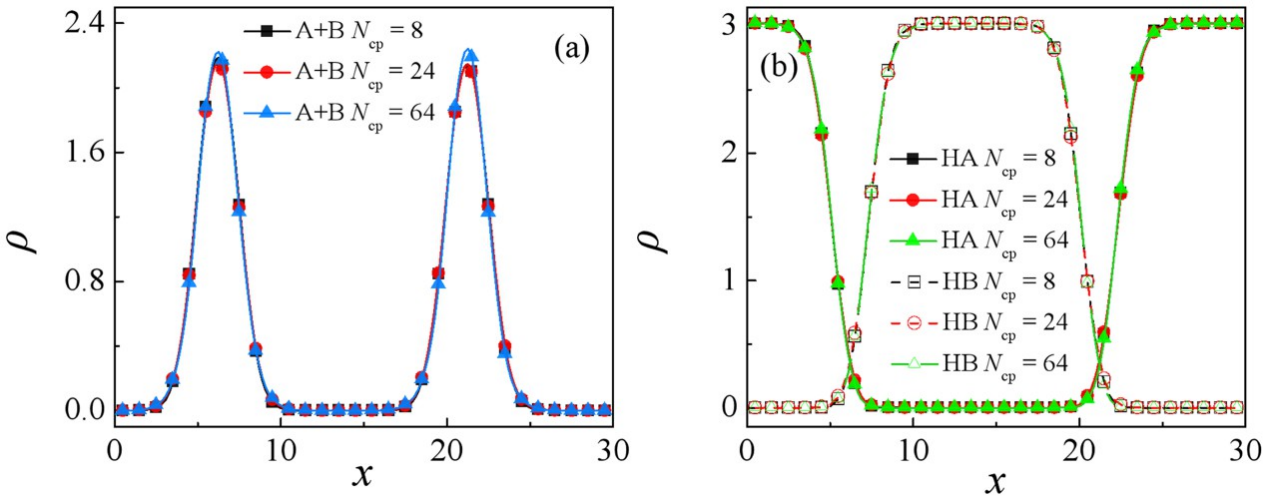
Density profiles of beads A and B of the (a) copolymers AB(*τ* = 8) and (b) homopolymers A_10_ and B_10_ in the *x*-direction as a function of the copolymer AB(*τ* = 8) chain length at *c*_cp_ = 0.15.

Fig 6 shows the relative density profiles of the copolymers AB (*τ* = 8) and the homopolymers A_10_ and B_10_ with copolymers chain length *N*_cp_ = 8, 24, 64. It is found that very little difference in the density profiles of the copolymers at various copolymer AB chain lengths is observed, as *N*_cp_ = 24, the densities of A+B beads of the copolymers AB (*τ* = 8) at the center of the interface are lower slightly [the red dots of Fig 6(a)], and as *N*_cp_ = 64, the density of A+B beads of the copolymers AB (*τ* = 8) at the center of the interface is higher slightly [the blue up triangles of Fig 6(a)]. However, little to no difference is observed in the density profiles of the A_10_ and B_10_ homopolymers at different copolymer chain lengths, as shown in Fig 6(b). This is because, despite the fact that the chain length of the AB copolymers is increased, the sequence length *τ* = 8 of copolymers remains unchanged. Hence, the density profiles of homopolymers at different copolymer chain lengths almost coincide.

**Fig 7:**
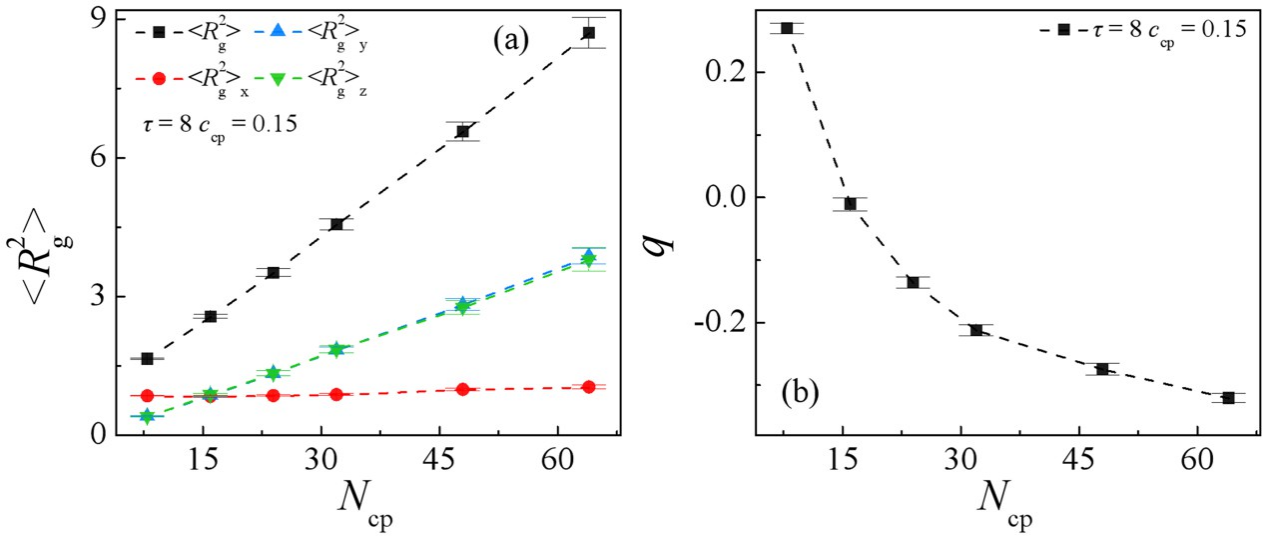
(a) Mean-squared radius gyration $\langle R_{g}{}^{2}\rangle$ and the three components ${\langle R_{g}{}^{2}\rangle }_{x}$, ${\langle R_{g}{}^{2}\rangle }_{y}$, ${\langle R_{g}{}^{2}\rangle }_{z}$, (b) orientation parameter of copolymer as a function of the copolymer AB(*τ* = 8) chain length at *c*_cp_ = 0.15. (*N*_cp_ = 8, 16, 24, 32, 48, 64).

Fig 7(a) and 7(b) show dependence of the mean-square radius of gyration $\langle R_{g}{}^{2}\rangle$ and its three components ${\langle R_{g}{}^{2}\rangle }_{x}$, ${\langle R_{g}{}^{2}\rangle }_{y}$, ${\langle R_{g}{}^{2}\rangle }_{z}$, and the orientation parameter *q* of copolymers on the copolymer AB(*τ* = 8) chain lengths. We found that as the chain length of the copolymers AB(*τ* = 8) increases from *N*_cp_ = 8 to 64, ${\langle R_{g}{}^{2}\rangle }_{x}$ almost remains unchanged, ${\langle R_{g}{}^{2}\rangle }_{y}$ and ${\langle R_{g}{}^{2}\rangle }_{z}$ increase monotonically, thus orientation parameter q decreases [Fig 7(b)]. Moreover, it is obvious that as *N*_cp_ = 8, ${\langle R_{g}{}^{2}\rangle }_{x}$ is larger than ${\langle R_{g}{}^{2}\rangle }_{y}$ and ${\langle R_{g}{}^{2}\rangle }_{z}$, the copolymer volume is shaped like a cylinder [16], as *N*_cp_ > 16, ${\langle R_{g}{}^{2}\rangle }_{x}$ = ${\langle R_{g}{}^{2}\rangle }_{y}$ = ${\langle R_{g}{}^{2}\rangle }_{z}$, whereas *N*_cp_ > 16, ${\langle R_{g}{}^{2}\rangle }_{x}$ is smaller than ${\langle R_{g}{}^{2}\rangle }_{y}$ and ${\langle R_{g}{}^{2}\rangle }_{z}$, the copolymer volumes are shaped like a pancake [16]. This means that when the sequence length of the copolymer *τ* = 8, with increasing the copolymer chain length, the volumes for the copolymer distributed at the interfaces vary from a cylinder shape to a pancake shape.

**Fig 8:**
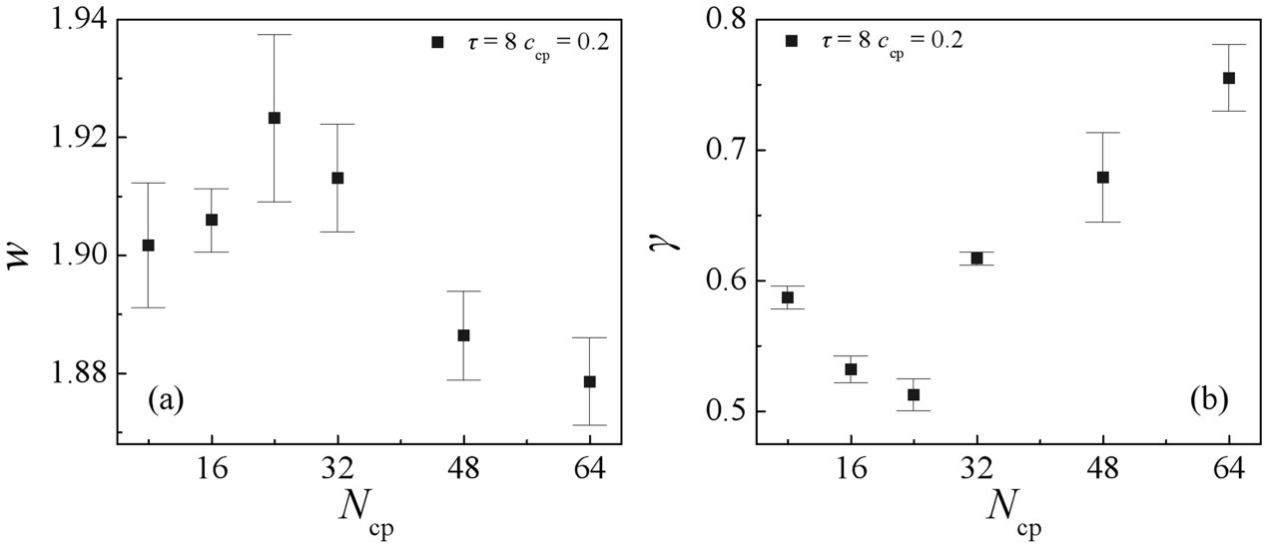
Interfacial width *ω* (a), and the interfacial tension *γ* (b) of the blends as a function of the copolymer AB(*τ* = 8) chain length at *c*_cp_ = 0.15. (*N*_cp_ = 8, 16, 24, 32, 48, 64).

Fig 8(a) and 8(b) depict the dependence of the interfacial width *ω* and the interfacial tension *γ* on the chain length *N*_cp_ of the copolymers AB(*τ* = 8). We found that although the density profiles of the copolymers and homopolymers at different copolymers chain lengths little change, the interfacial width *ω* and interfacial tension *γ* vary significantly. Specifically, as the copolymers’ chain length increases from *N*_cp_ = 8 to 24, the interfacial width *w* increases [Fig 8(a)], and the interfacial tension *γ* decreases [Fig 8(b)]. This result is inconsistent with the results of diblock and triblock copolymers, in a blended system of diblock or triblock copolymers, the shorter the chain length of the diblock and the triblock copolymer the lower the interfacial tension [24, 28]. As the chain length of the sequenced copolymer further increases from *N*_cp_ = 24 to 64, the interfacial width *w* decreases the interfacial tension increases. These results indicate that the blends composed of the copolymers AB(*τ* = 8) with the chain length *N*_cp_ = 24 displays a wider interfacial width and a lower interfacial tension than those of other chain lengths. Therefore, we conclude that the copolymers AB(*τ* = 8) with the chain length *N*_cp_ = 24 serve as more effective compatibilizers than the short diblock and the long sequence copolymer for strengthening the interfaces. S2 Fig shows the morphology snapshots for the blends of A_10_/AB(*τ* = 8)/B_10_ of different copolymer chain lengths, respectively. We found that as the chain lengths of copolymers increase from *N*_cp_ = 8 to 64, the interfaces undergo the following changes: i) flat interfaces of not saturation (*N*_cp_ = 8 and 16), ii) interface saturation with change in the interfacial geometry (*N*_cp_ = 24 and 32), iii) formation of micelles (*N*_cp_ = 48 and 64). Therefore, we inferred that the reason of the nonmonotonic behavior of the interfacial tension and the interfacial width in Fig 8 might be the no saturation for *N*_cp_ < 24 and the over saturation for *N*_cp_ > 24.

## Conclusion

In this paper, the DPD simulation method is used to explore the effects of the concentration and the chain length of sequence copolymer with sequence length *τ* = 8 on the interfacial properties of A_10_/AB(*τ* = 8)/B_10_ ternary blends.

Our simulations show that as the concentration of the copolymer AB(*τ* = 8) (*N*_cp_ = 32) increases from *c*_cp_ = 0.05 to 0.15, the density of beads A and B of copolymer AB(*τ* = 8) at the interface significantly increases, which results in the reduction of the interfacial tension. However, as *c*_cp_ of the AB(*τ* = 8) copolymers further increases from *c*_cp_ = 0.15 to 0.2, due to the copolymers AB(*τ* = 8) at the interface has formed micelles at *c*_cp_ = 0.2, and the micellization could lower the efficiency of the copolymers as a compatibilizer, therefore, the interfacial tension increases slightly. By elevating the chain length of the sequence copolymer AB(*τ* = 8) from *N*_cp_ = 8 to 64 at *c*_cp_ = 0.15, the copolymer volumes vary from a cylinder shape to a pancake shape. As the chain length of the copolymer AB(*τ* = 8) *N*_cp_ = 24, the blend exhibits a wider interfacial width *ω* and a lower interfacial tension, which indicates the copolymers AB(*τ* = 8) of the chain length *N*_cp_ = 24 for strengthing the interface is more prominent.

The obtained results indicate that the structural and mechanical properties of the blends composed of the sequenced copolymer are strongly correlated to the concentration and the chain length of the copolymers. In this context, we deduce it would be necessary to further investigate the dependence of the interfacial and phase behaviors of the ternary blend composed of sequence copolymer on the other molecular parameters.

## Supporting information

S1 Fig Representative morphology snapshots for A_10_/AB(*τ* = 8)/B_10_ ternary at different simulation times. Compositions are A_10_/AB(*τ* = 8)/B_10_ ternary blends for (a1, a2), and AB (*τ* = 8) copolymers for (b1, b2). Chain length and concentration of the copolymer are fixed as *N*_cp_ = 32, *c*_cp_ = 0.2. Red and yellow spheres represent bead A and bead B of homopolymers, and green and blue spheres represent beads A and B of the copolymers.
(PDF)

S2 Fig Morphology snapshots of systems at different copolymer chain length. (a)A_10_/AB(*τ* = 8)/B_10_ ternary blends and (b) AB (B_10_) copolymers at *c*_cp_ = 0.15. The red and yellow spheres denote bead A and bead B of homopolymers A_10_ and B_10_, and the green and blue spheres represent beads A and B of the AB copolymers.
(PDF)
