# Supplementary figures and images for: Effects of concentration and chain length of the sequence copolymer on interfacial properties of homopolymers/sequence copolymers ternary blends: A DPD simulation study
Source: PLoS One. 2022 Jul 26;17(7):e0270094. doi: 10.1371/journal.pone.0270094 (PMC9321409; doi:10.1371/journal.pone.0270094)

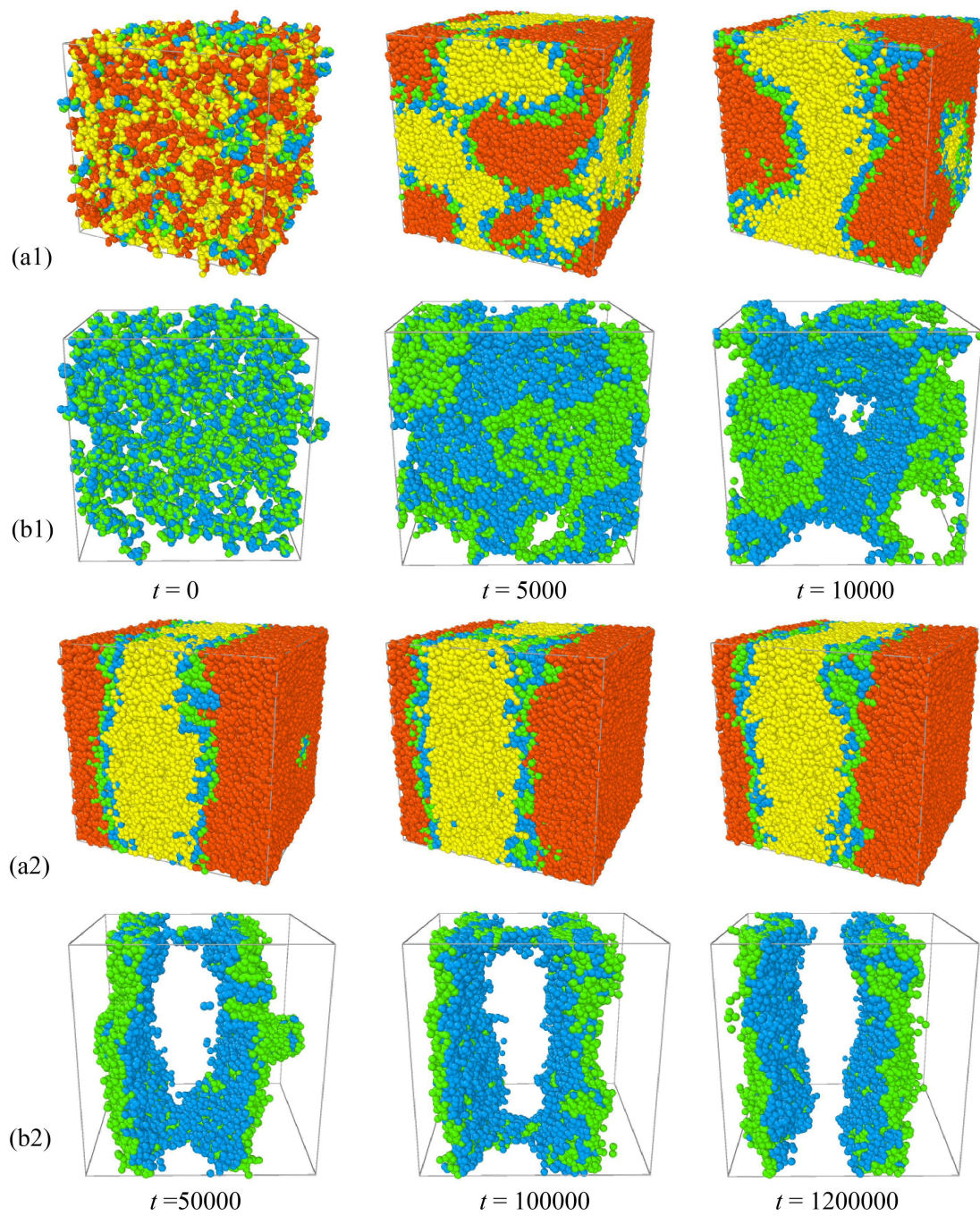

Supplement: S1 Fig — Compositions are A10/AB(τ = 8)/B10 ternary blends for (a1, a2), and AB (τ = 8) copolymers for (b1, b2). Chain length and concentration of the copolymer are fixed as Ncp = 32, ccp = 0.2. Red and yellow spheres represent bead A and bead B of homopolymers, and green and blue spheres represent beads A and B of the copolymers. (PDF) [file pone.0270094.s001.pdf]

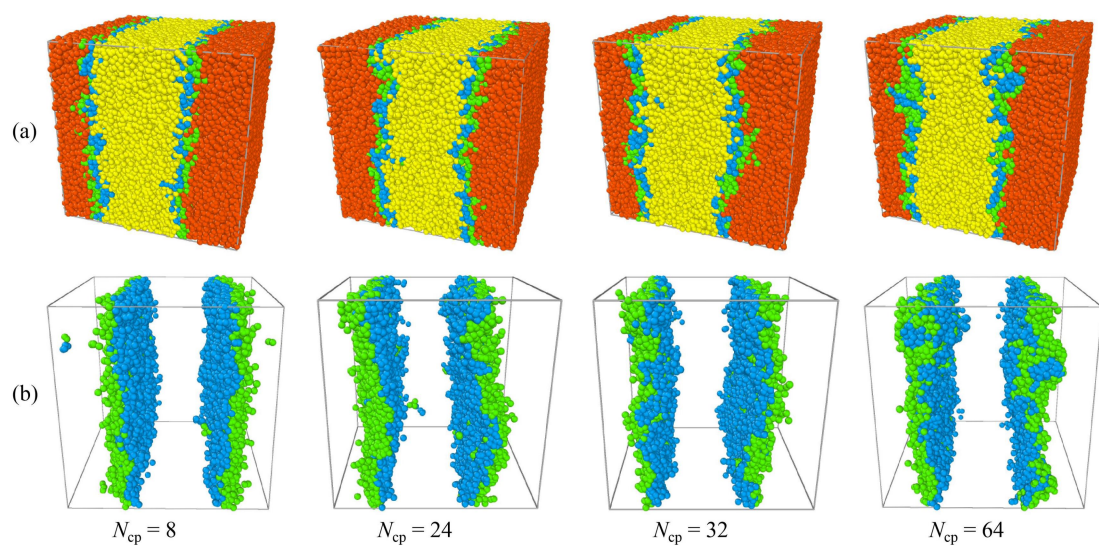

Supplement: S2 Fig — (a)A10/AB(τ = 8)/B10 ternary blends and (b) AB (B10) copolymers at ccp = 0.15. The red and yellow spheres denote bead A and bead B of homopolymers A10 and B10, and the green and blue spheres represent beads A and B of the AB copolymers. (PDF) [file pone.0270094.s002.pdf]
